# Microwave Synthesis of Au Nanoparticles in the Presence of Tetrahydrothiophenocucurbituril

**DOI:** 10.3390/molecules29010168

**Published:** 2023-12-27

**Authors:** Asma S. Atthar, Shreya Saha, Ahmed Abdulrahman, Anthony I. Day

**Affiliations:** Chemistry, School of Science, University of New South Wales Canberra, Australian Defence Force Academy, Canberra, ACT 2600, Australia; asmasamaunnisa@gmail.com (A.S.A.); shreya.saha@adfa.edu.au (S.S.); a.abdulrahman@adfa.edu.au (A.A.)

**Keywords:** Au nanoparticles, tetrahydrophenocucurbituril, tetrahydropheno reductant, formic acid reductant, gold(I)

## Abstract

The preparation of gold nanoparticles (AuNPs) from tetrachloroauric acid in the presence of tetrahydrothiophenocucurbit[*n*]uril (THT*_m_*Q[*n*]) has been effectively achieved in a microwave reactor. The reaction was performed in the presence of an excess of the tetrahydrothiopheno function in a partial reductant role, while the remainder formed AuNP-THT*_m_*Q[*n*] conjugates after the reduction was completed with formic acid. An affinity for the AuNPs by the THT*_m_*Q[*n*] was observed in the purification of the NPs via centrifugation, removal of the supernatant and resuspension of the conjugate.

## 1. Introduction

The preparation and stabilization of gold nanoparticles (AuNPs) has captured the imagination of many, especially in their potential for applications in biotechnology, which includes biosensors, drug delivery and therapies [1,2,3,4,5,6,7,8]. A collection of synthetic methods has been reported for the preparation of AuNPs which involve a conventional chemical reduction [9,10,11], activated reduction through photolytic [12,13], sonochemical [14,15], or an amine-stabilized microwave method [16]. Also, some of these methods are assisted by seed growth techniques for size and morphological control [12,17,18]. One of the first examples of this technique involved the seeding of solutions with smaller Au particles to act as nucleation sights to form larger uniform particles with UV activation in the presence of TX-100 (a reducing and stabilizing agent) [12]. Another method involved reduction with hydroquinone/citrate in different ratios in the presence of seed AuNPs (40 nm) to form large reproducible particles of up to 100 nm [17,18]. In addition to the pure AuNPs, there are developments toward Au nanohybrids, where AuNPs are coated with a second or third metal layer such as Co and Fe. Functionalized Au nanohybrids of this type can serve in applications of diagnostics, sensing, and drug delivery [8,19]. 

One relatively recent area of interest is the preparation of AuNPs in the presence of the macrocyclic host molecule cucurbit[*n*]uril (Q[*n*]) [20,21,22,23,24,25,26]. This family of macrocycles are of particular interest due to their excellent molecular host–guest properties, which are potentially applicable to the construction of sensors [22,23,27,28], catalysts [21,23,29] and drug delivery vehicles [24,25,30], particularly as AuNP conjugates. The preparation of AuNP-Q[*n*] conjugates has been demonstrated by several methods involving an in situ reduction of Au salts in the presence of Q[*n*]. A direct approach is NaBH_4_ reduction in the presence of Q[*n*] in water [31,32,33], or indirectly involving the Brust–Schiffrin method to prepare thiol derivatives attached to AuNP, that were then solubilized in water in the presence Q[*n*], where part of the thiol derivative becomes encapsulated [24]. Citrate-reduced and -stabilized AuNP were also added to aqueous solutions of Q[*n*] [28]. In addition, AuNP-Q[*n*] were prepared where curcumin was the reducing agent in the presence of Q[7] [34] or where an aqueous solution of hydrogen peroxide was the reductant [29]. A report of Q[7] and Au salts dissolved is aqueous NaOH (40 mM) also led to AuNP-Q[7] without a clear explanation for the source of the reducing species [21]. Each of these methods have a useful place in the preparation of AuNP-Q[*n*] conjugates. However, an intended application for AuNP-Q[*n*] may be compromised by residual reduction by-products such as borate salts or oxidized organic material, which can limit the molecular encapsulation capacity of the cavity of Q[*n*]. Therefore, purification can be an important consideration for the use of AuNP-Q[*n*] for sensitive applications [23,27,28].

We report a novel example for the in situ synthesis of AuNP-Q[*n*] conjugates where some of the reductant is a constituent of the Q[*n*] as a tetrahydrothiophene (THT). The equatorial location of the THT substituent also potentially provides an Au attachment point, leaving the cavity free for guests. Formic acid was also found to be an important reducing component in this process.

**Figure 1:**
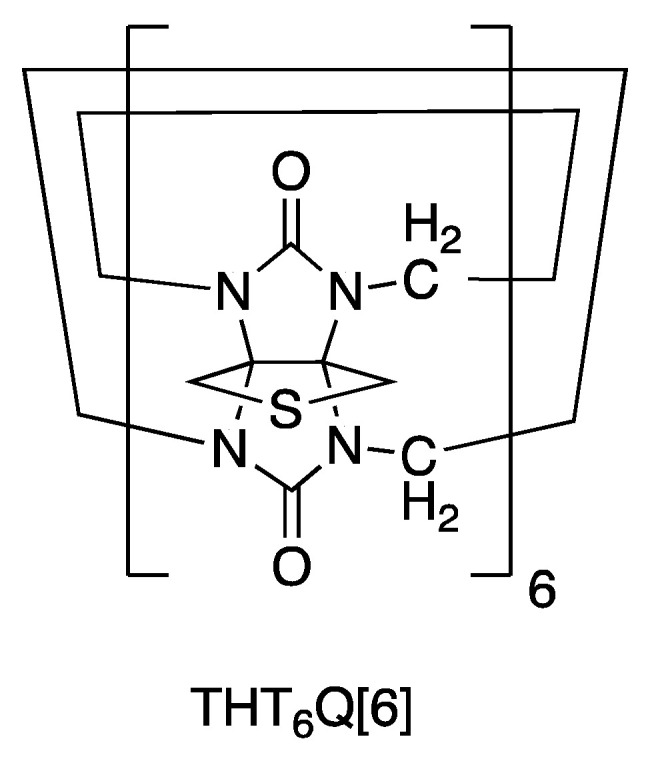
Tetrahydrothiophenocucurbit[6]uril (THT_6_Q[6]).

Recently, we reported the synthesis of tetrahydrothiophenocucurbit[6]uril (THT_6_Q[6], Figure 1) and the preparation of AuNP conjugates with this macrocycle [33]. In the previous report, AuNPs were prepared in the presence of THT_6_Q[6] where tetrachloroauric acid (HAuCl_4_) was reduced with NaBH_4_, yielding AuNP-THT_6_Q[6]. Initially, these conjugates were dispersed particles which slowly (weeks) formed chains of particles. The NaBH_4_ reduction method is fast and convenient, although to obtain clean and functional AuNP-THT_6_Q[6] conjugates, a purification process is required to remove undesirable salts [35]. Our interest in the preparation of AuNPs as THT*_n_*Q[*n*] conjugates led us to consider an alternative approach to the reduction of Au(III) to form NPs with fewer by-products.

The pertinent synthesis of a tetrahydrothiopheneAu(I) chloride complex ([Au(SC_4_H_8_)]Cl) was reported in 1989 from a reaction of HAuCl_4_ in the presence of 2 equiv. of tetrahydrothiophene (SC_4_H_8_). In this reaction, Au(III) was reduced to Au(I), which reacts with 1 equiv. of SC_4_H_8_ to form the complex [Au(SC_4_H_8_)]Cl, and the remaining 1 equiv. acts as the reductant giving tetrahydrothiopene-1-oxide [36]. While this did not form AuNP, it was only one electron away from Au(0). Therefore, we considered an approach to achieving this last step by heating in a microwave reactor (MW). Reactions performed in an MW are known to be accelerated, have heat homogeneity and generally produce higher yields of products.

## 2. Results and Discussion

**Scheme 1:**
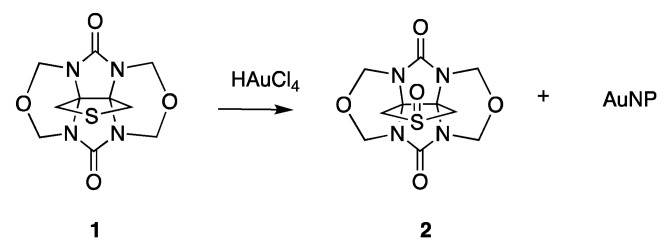
The MW reaction of THTG **1** in a mixture of DMSO/water (2:1) producing sulfoxide **2** and AuNPs.

In the first instance, we used the readily available tetrahydrothiophenoglycoluril diether derivative THTG **1** as a model. THTG **1** has been shown to have similar chemical reactivity to the THTG moiety in THT_6_Q[6], as **1** is a synthesis precursor to this macrocycle [33]. Interestingly, when we applied this reaction to THTG **1**, in a mixture of DMSO/H_2_O in a microwave reactor at 70 °C, not only was the sulfoxide **2** obtained, but also AuNPs with no observed Au(I) complexes (Scheme 1). Repeating the reaction in H_2_O alone still formed AuNPs, although the quantity was smaller primarily due to the poor aqueous solubility of **1**. AuNPs were formed in the size range of 5–10 nm, consistent with the visible spectrum λ_max_ = 523 nm (Figure S5).

**Scheme 2:**
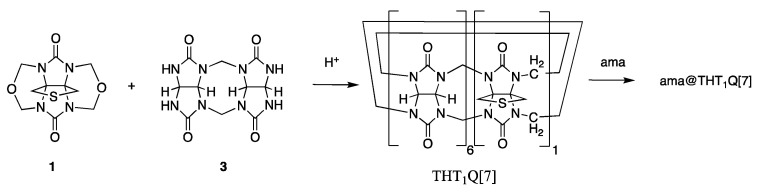
The synthetic approach to the preparation of monosubstituted tetrahydrothiophenocucurbit[7]uril (THT_1_Q[7]), isolated as ama@THT_1_Q[7], Cl or PF_6_ salt.

The evaluation of this reaction was then extended to THT-substituted Q. THT_1_Q[7], a partially substituted Q[7], was prepared from an acid catalyzed condensation reaction of THTG **1** and unsubstituted glycoluril dimer **3** (Scheme 2). The purification of THT_1_Q[7] was facilitated by the encapsulation of amantadine HCl (ama) to form the ama@THT_1_Q[7] salt and cation exchange resin chromatography. The ama@THT_1_Q[7] association complex provided a water-soluble THTQ derivative carrying only a single THTglycoluril moiety suitable for the evaluation of the THT redox reaction with Au(III) in the MW reactor. The conditions applied were similar to those described above, except that no organic solvent was needed. The ama@THT_1_Q[7] association complex was preferred as a study candidate in the first instants based on the simplification of the reaction with only a single THTglycoluril moiety.

### 2.1. AuNP-ama@THT_1_Q[7]

**Figure 2:**
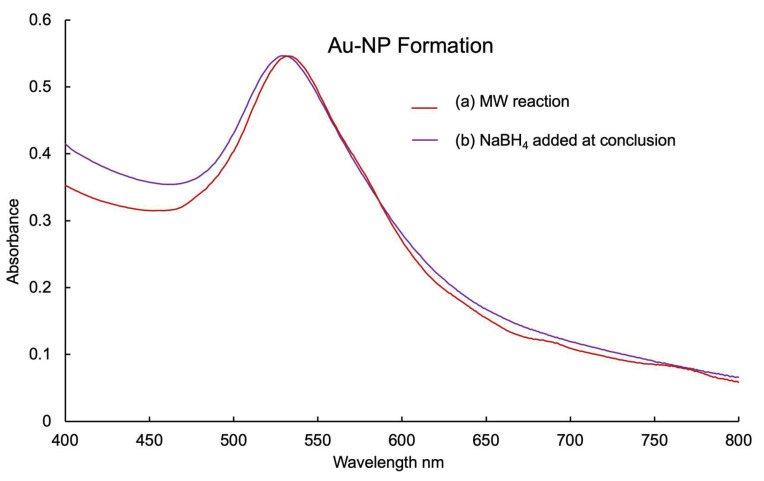
The visible spectra of the MW reaction 15 min at 70 °C, 3:1 ama@ THT_1_Q[7] to HAuCl_4_ (a) followed by the addition of NaBH_4_ (b).

An aqueous solution of ama@THT_1_Q[7]Cl combined with HAuCl_4_ in a mole ratio of 1:1 heated in the MW reactor at 70 °C for 10 min produced AuNPs, evident by the red-blue color of the solution, which was supported by the visible spectrum (λ_max_ = 535 nm, Figure S6). An addition of an aqueous solution of NaBH_4_, a demonstrated method for the formation of AuNPs from HAuCl_4_, increased the absorbance by 24%, with a small blue shift to λ_max_ = 526 nm (Figure S6) [31,32,33]. This indicated that not all of the Au salts were reduced to Au(0). Repeating the reaction with an increased ratio of 2:1 (ama@ THT_1_Q[7] to HAuCl_4_) over a 15 min period resulted in an absorption band at λ_max_ = 538 nm and the NaBH_4_ test only led to an 11% increase at λ_max_ = 539, slightly red-shifted (Figure S7). Finally, at a ratio of 3:1 at 70 °C for 15 min, the result was the complete reduction of the Au salts. At this higher ratio, no increase in the absorbance occurred after the addition of NaBH_4_ to the reaction mixture (Figure 2, curves a and b, respectively). These curves are almost superimposable.

**Scheme 3:**
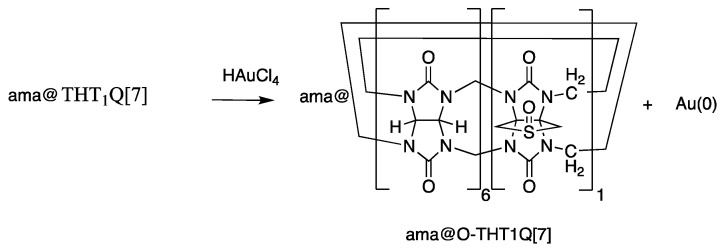
The MW reaction of ama@THT_1_Q[7] and HAuCl_4_ in water at 70 °C for 15 min.

Given that the THT functionality was expected to be the reductant with the formation of a sulfoxide, this was anticipated to be evident with the formation of ama@O-THT_1_Q[7] (Scheme 3). New resonances were observed in the ^1^H NMR spectra of the MW reaction mixtures as multiplets between 4.70 and 4.45 and a doublet at 3.47 ppm.

The formation of the sulfoxide ama@O-THT_1_Q[7] was verified by preparing the same compound by separately oxidizing ama@ THT_1_Q[7] with NaIO_4_ in water to produce a high yield of ama@O-THT_1_Q[7]. Purified ama@O-THT_1_Q[7] showed two distinguishable resonances as two doublet proton resonances at 4.66 and 3.48 ppm (J = 14.7 Hz), for the α CH_2_ relative to the sulfoxide (Figure S2). The downfield-shifted resonance was consistent with 2H on the same face as the S=O group on the glycoluril moiety and the remaining resonance (3.48 ppm) for the 2H on the opposite face, respectively. These resonances were similar to the coupling and downfield shift for equivalent protons of the glycoluril sulfoxide **2** [33].

However, given that the stoichiometry for the reaction, as depicted in Equation (1), 1.5 moles of ama@THT_1_Q[7]Cl should have been sufficient to reduce 1 mole of HAuCl_4_. The higher ratio of ama@THT_1_Q[7]Cl to HAuCl_4_ of 3:1 leading to the complete reaction was initially difficult to explain. This was also inconsistent with the relative integral values between the remaining THT singlet proton resonance at 3.56 ppm and the formed sulfoxide doublet proton resonance at 3.48 ppm (integral value 2:1).

3(ama@THT_1_Q[7]) + 2Au(III) + 3H_2_O → 3(ama@O-THT_1_Q[7] + 2Au(0) + 6H^+^ (1)

It was observed that some samples of ama@THTQ[7]Cl and HAuCl_4_ at the same mole ratio of 3:1 were found to have substantially slower reaction times than others, even though the final results were the same. It was also observed that after 30 min of the MW reaction, the reaction continued slowly at room temperature over days, as was evident by an increase in the intensity of the colored AuNP solution.

Interestingly, when a purified ama@THT_1_Q[7]PF_6_ salt was used, also at a ratio of 3:1 (Q:Au(III)), the result was different. Adding the HAuCl_4_ solution to the ama@THT_1_Q[7] PF_6_ gave a slightly cloudy pale-yellow mixture, which was identical to ama@THT_1_Q[7]Cl samples. However, in the case of the PF_6_ salt, the conditions of 15–30 min at 70 °C in the MW reactor produced a colorless clear solution. A ^1^H NMR spectrum of this solution showed that the THT protons were broad, the sulfoxide doublet was evident and relatively sharp, and there were no other linewidth changes in the other peaks (Figure S4). This suggested that the first step of the reaction was as depicted in Equation (2). Half of the ama@THT_1_Q[7] was oxidized, while the remainder formed the ama@THT_1_Q[7]Au(I) complex.

2(ama@THT_1_Q[7]) + Au(III) + H_2_O → ama@O-THT_1_Q[7] + ama@THT_1_Q[7]Au(I) + 2H^+^ (2)

The final reduction to Au(0), hence AuNP, was then reasonably suspected of involving formic acid as the reducing agent. Formic acid was found as a minor impurity in the samples of ama@THT_1_Q[7]Cl, but was absent in the ama@THT_1_Q[7]PF_6_ sample. This was tested by the addition of 1 mole equiv. of formic acid after the conclusion of the first stage of the reaction, which gave a red solution with a λ_max_ = 528 nm after heating in the MW reactor. Therefore, equation 3 depicts the last step in the reduction to AuNPs.

ama@THT_1_Q[7]Au(I) + 0.5HCO_2_H → ama@THT_1_Q[7]Au(0) + H^+^ + 0.5CO_2_ (3)

**Figure 3:**
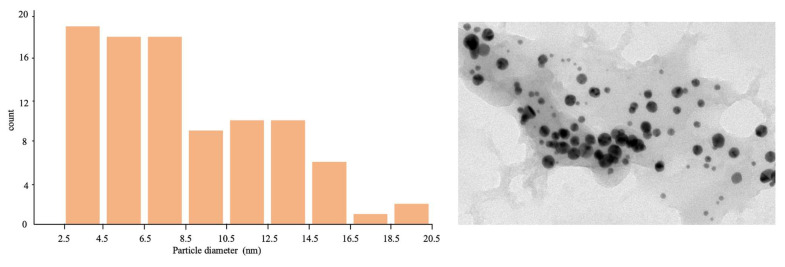
TEM micrograph (RHS) and size distribution histogram (LHS) for a sample count of 93 AuNPs.

Centrifugation of the reaction solution of AuNP-ama@THT_1_Q[7] and removal of the supernatant gave a plug that was suspended in D_2_O. Compared to water and at the same concentration, the stability of AuNP-ama@THT_1_Q[7] was identical. The ^1^H NMR spectrum of this solution showed resonances consistent with those of ama@THT_1_Q[7]; however, no resonances were present for the sulfoxide. These purified AuNP-ama@THT_1_Q[7] samples were examined employing TEM imaging, which showed a distribution of particle sizes of 2.5–16.5 nm. The dominant size range was between 2.5 and 8.5 nm and a second group in the range of 8.5–16.5 nm (Figure 3). The smaller size range constituted ~60% of the particle count. The particles were relatively freely dispersed (Figure S8).

The absence of the sulfoxide (ama@O-THT_1_Q[7]) in purified samples was unexpected given that AuNPs are reported to associate with Q portals [22,27,31]. The affinity for association was independently examined by preparing AuNPs via the conventional method using NaBH_4_, purifying them via centrifugation and then with sonication, resuspending them in a solution of ama@O-THT_1_Q[7]Cl. When these samples were purified via centrifugation and resuspension in D_2_O, the ^1^H NMR spectra also revealed the absence of resonances for ama@O-THT_1_Q[7]Cl. From these results, we conclude that the affinity of ama@O-THT_1_Q[7]Cl for the AuNPs must be low.

Significantly, this result demonstrates that some of the added ama@THT_1_Q[7] is sacrificed as the reducing agent for HAuCl_4_, and that the oxidized portion, ama@O-THT_1_Q[7], can be easily removed via centrifugation while maintaining the Au(0) association with the unreacted ama@THT_1_Q[7].

The importance of the THT functional group was also verified by applying identical reaction conditions to unsubstituted Q[7] and ama@Q[7]. For both examples, no AuNPs were formed in the MW reactor, even after the addition of formic acid.

The purification of THT*_m_*Q[*n*] samples primarily relies on the separation of Dowex cation exchange resin and the eluant that is the most effective in aqueous formic acid. Residual formic acid can therefore be found in highly purified samples, which was found to be an important reductant in the last step of the preparation of AuNP-THT*_m_*Q[*n*] conjugates during the MW reaction. Its importance was also demonstrated in the example discussed in the next section.

### 2.2. AuNP-THT6Q[6]

Applying the MW reactor conditions to a fully substituted example, THT_6_Q[6] (Figure 1) suspended in an aqueous solution of HAuCl_4_ in a mole ratio of 1:1 heated in the MW reactor at 70 °C for 15 min produced a faint red color, indicating the formation of AuNPs. Repeating the reaction for 15 min gave an increase in the intensity of color, but Au salts remained unreacted, as well as suspended THT_6_Q[6]. The solubility of THT_6_Q[6] in pure water is <200 μM. Adding THT_6_Q[6] to saline improved the solubility but decreased the performance toward AuNP formation with only a very faint color.

The preparation of the association complex of cyclopentylammoniun salt (cpn) with THT_6_Q[6], as previously described [33], improves the aqueous solubility to 0.8 mM. The cpn@THT_6_Q[6] complex subjected to the same conditions above led to only a faint red color. Adding formic acid, as described in Section 2.1, and a repeat of the heating in the MW reactor gave an intense red color at λ_max_ = 241 nm. This wavelength is consistent with the formation of AuNP-THT_6_Q[6] previously reported for the NaBH_4_ reduction method [33]. The presence of multiple THT groups per macrocycle influences the aggregation of the AuNPs.

THT_6_Q[6] also has acceptable solubility of 1 mM in aqueous Ca(OAc)_2_ (50 mM). When an aqueous solution of HAuCl_4_ was added to a mole ratio of 1:1 and heated in the MW reactor at 70 °C for 15 min, there was a complete formation of AuNP. The formed red solution was found to be stable with a visible spectrum absorption band at λ_max_ = 527 nm (Figure S8). The ^1^H NMR spectrum of a freeze-dried sample clearly showed resonances for THT_6_Q[6]; however, proton resonances for the evidence of sulfoxide formation was inconclusive due to these peaks being obscured by the THT_6_Q[6] resonances. This is consistent with the reported chemical shifts of the sulfoxide of THT_6_Q[6] [33]. It was also noted that the lack of visibility of the sulfoxide proton resonances is likely further complicated by the fact that six THT groups were present for every mole of THT_6_Q[6] and on average only one will be oxidized. Hence, the remaining THT proton resonances would overwhelm the sulfoxide proton resonances. With an initial concentration of THT_6_Q[6] of 1 mM for this reaction, the acidity of the solution at the conclusion was a pH of 5.5 at room temperature.

Subjecting the AuNP-THT_6_Q[6] solution to centrifugation gave a dark-colored plug. Removal of the supernatant liquid and the addition of D_2_O with sonication returned the AuNP-THT_6_Q[6] to its suspended state. A ^1^H NMR spectrum of this solution showed the presence of THT_6_Q[6] plus some remaining acetate. Repeating the process removed more acetate but not completely.

It is noteworthy that the visible spectrum (Figure S9) of the AuNP-THT_6_Q[6] prepared here had a close fit to our previous reported preparation of AuNP-THT_6_Q[6] employing the conventional NaBH_4_ method. However, in that reported example, the fit was only relevant to the reaction performed on a solution with a low ratio of THT_6_Q[6] to Au(III) [33].

## 3. Materials and Methods

^1^H NMR spectra were recorded at 25 °C at 400 MHz on a Varian (Las Vegas, NV, USA) Unityplus-400 spectrometer, as specified in D_2_O, and were referenced using the residual HDO resonance at 4.78 ppm. Concentrations were determined by comparing the integrals of the THT_m_Q[*n*] samples with known concentrations of sodium benzoate in D_2_O as an internal standard. The pulse repetition was delayed for 20 s for these determinations. A Biotage (Uppsala, Sweden) Initiator+ EU 356006 Microwave Reactor was used with 0.5–2 mL clamp-sealed microwave reaction vessels. Auto-controlled microwave power output maintained a constant temperature at 70 °C for a constant time. On average, the microwave power was 14–20 W for most of the reaction period. Low- and high-resolution mass spectrometric analysis using the ESI (Pittsburgh, PA, USA) (TOF) technique was performed using a Waters (Milford, MA, USA) Synapt G2-Si HDMS mass spectrometer equipped with a Z-spray/Lockspray ESI/APCI/ESCi source coupled to an Acquity 3000 UPLC I class plus (Waters). Infrared spectra were generated on a Shimadzu (Tokto, Japan) IRPrestige 21 FTIR spectrometer with samples analyzed with 16 scans through the 700–4000 cm^−1^ window with 4 cm^−1^ resolution, as KBr discs. UV-Vis studies were performed on a Varian Cary (Cary, NC, USA) 500 Bio UV-Vis spectrophotometer from 400 to 800 nm in water.

TEM characterization was performed using an electron microscope unit at the Mark Wainwright analytical center, University of New South Wales, Sydney, using an FEI Tecnai G2 20 TEM.

All purchased chemicals and reagents, such as tetrachloroauric acid (HAuCl4), sodium borohydride (NaBH_4_), ammonium hexafluorophosphate (NH_4_PF_6_), calcium acetate ((Ca(OAc)_2_), amantadine HCl (ama) and cyclopentamine, were obtained from Sigma-Aldrich, while D_2_O was obtained from Cambridge Isotope Laboratories. All solvents and reagents were used as provided and aqueous solutions were made using Milli-Q water from a Millipore four-stage water purification unit.

### 3.1. Synthesis of AuNP-THTglycoluril

A DMSO solution of THTglycoluril (5.0 mL, 0.27 mM) was added dropwise to an aqueous solution of HAuCl4 (2.5 mL, 0.13 mM) with stirring in a vial to give a clear yellow-colored solution. The resulting mixture was placed in the MW reactor chamber set to 70 °C, with stirring for 10 min (two 5 min cycles) to afford the AuNP solution. The glycoluril sulfoxide was spectroscopically identical to that previously reported [33].

### 3.2. Synthesis of Amantadinylammonium@tetrahydrothiophenoQ[n]Cl (ama@THT_1_Q[7]Cl)

Glycoluril dimer [37] (2.0 g, 6.5 mmol), tetrahydrothiophenoglycoluril diether **1** (932 mg, 3.25 mmol) and LiCl (123 mg) were added together and ground to form a homogenous mixture. To this solid mixture, 32% conc. HCl (8 mL) was added and warmed to 40 °C, with stirring for 1 h. An additional portion of a finely powdered glycoluril dimer (1.0 g, 3.25 mmol) was added. When all solids dissolved, paraformaldehyde (390 mg, 13.0 mmol) in 4 portions was added over 40 min. The temperature was then increased to 90 °C and stirred for 12 h. After cooling, HCl and water were evaporated on a rotary evaporator, leaving a brown solid material (3.3 g). Amantadine hydrochloride (ama) (180 mg) was added to the crude product dissolved in a minimum volume of 50% formic acid solution. This was loaded onto a column of Dowex 50WX2 cation exchange resin and the products were eluted with 0.5 M HCl/50% formic acid. Fractions were continuously monitored by ^1^H NMR to identify the elution of fractions rich in ama@THTQ[7]. The fractions abundant in THT_m_Q[7] were combined and the chromatography repeated to remove any unsubstituted Q[7]. The best fractions were combined, and the solvent evaporated to give a colorless solid residue. This solid was then boiled in a minimum volume of water, where almost all solid dissolved. After cooling, the solution was filtered to remove any insoluble material. A saturated solution of NH_4_PF_6_ was then added dropwise until no more turbidity occurred. The turbid solution was heated and allowed to cool. The solid was collected and after repetitive crystallization from water 370 mg of pure, ama@THT1Q[7]PF_6_ salt was obtained. On a short Dowex 50WX2 column, anion replacement was achieved by dissolving ama@THT1Q[7]PF_6_ in 50% formic acid water and loading onto the column. The subsequent eluant was 50% formic acid/ 0.5 M HCl initially, and then, this was increased to 0.6 M HCl. The collected chloride form was then used for subsequent reactions.

Mp > 320 °C. IR (KBr, cm^−1^): 3007 w, 2972 m, 2857 w, 1737 s, 1476 s, 1424 m, 1378 s, 1317 s, 1253 m, 1232 s, 1191 s, 1154 m, 1028 w, 988 m, 969 s, 921 m, 852 m, 827 m, 806 s, 757 m. ^1^H NMR (400 MHz, D_2_O): δ 5.82–5.70 (m, 14H), 5.55 (bs, 12H), 4.38–4.22 (m, 14H), 3.56 (s, 4H); [1.44 (bs, 3H), 1.19 (d, 3H, *J* = 12 Hz), 1.15 (s, 6H), 0.87 (d, 3H, *J* = 12 Hz)—amantadinyl resonances]. MS (ESI) *m/z*: 686.7 [amantadinylNH_3_^+^@ THTQ[7] + H^+^]^2+^/2. Anal calc. for C_54_H_61_N_29_O_14_S.17H_2_O: C, 37.82; H, 5.64; N, 23.69; S, 1.86. Found: C, 37.81; H, 5.63; N, 23.46; S, 1.83.

### 3.3. Synthesis of Amantadinylammonium@tetrahydrothiopheno-1-oxideQ[n] (ama@O-THT1Q[7]Cl)

Ama@THT1Q[7]Cl (20.0 mg, 0.014 mmol) was added to water (10 mL), sonicated and heated alternatively to dissolve all the solids. The solution was then syringe-filtered to remove any insoluble solids present (majority of the solid was solubilized due to high volume of water). To this clear solution, NaIO_4_ (3.0 mg, 0.014 mmol) was added which led to temporary turbidity. The reaction was maintained at RT for 52 h. The mixture was freeze-dried, and the residue suspended in a minimum volume of water and loaded onto a small column of Dowex 50WX2 cation exchange resin. The product was eluted with 0.5 M HCl/50% formic acid.

Mp > 320 °C. IR (KBr, cm^−1^): 3018 w, 2942 w, 2852 w, 1737 m, 1474 m, 1402 w, 1321 s, 1236 s, 1196 s, 1156 m, 1050 w, 1028 w, 968 s, 805 m, 757 s. 1H NMR (400 MHz, D_2_O): δ 5.88–5.71 (m, 14H), 5.61–5.50 (m, 12H), 4.62 (d, 2H, *J* = 14.7 Hz), 4.52 (d, 2H, *J* = 15.5 Hz), 4.47 (d, 2H, *J* = 15.5 Hz), 4.37–4.18 (m, 12H), 3.45 (d, 2H, *J* = 14.7 Hz), 1.45 (bs, 3H), 1.24–1.12 (m, 9H), 0.87 (d, 3H, *J* = 12.4 Hz). HRMS (ESI-TOF) *m*/*z*: [ama@O-THT1Q[7]] + calcd for C_54_H_62_N_29_O_15_^32^S 1388.4701. Found: 1388.4701.

### 3.4. Reaction Procedure for the Microwave Reactor

#### 3.4.1. Preparation of AuNP-ama@THT_1_Q[7]Cl

A stock solution of ama@THT_1_Q[7]Cl was prepared at a concentration of 1.6 mM. In addition, a stock solution of HAuCl_4_ was prepared with a concentration of 10 mM and of NaBH_4_ in water (0.1 M). The concentration of THT_1_Q[7] samples were determined using an internal reference of standardized sodium benzoate (10 μL, 5.3 mM D_2_O) in a D_2_O solution (500 μL) of each relevant sample.

A. Mole ratio 1:1 (THTQ:Au(III))

The ama@THT_1_Q[7]Cl (652 μL) was added to H_2_O (1275 μL), and to this, a HAuCl_4_ solution (100 μL) was added. The slightly turbid solution was placed in the MW reactor and stirred at 70 °C for 5 min, and then repeated up to an additional 5 min with no significant difference in the visible absorption spectrum at λ_max_ = 535 nm. The remaining Au salts were verified by the addition of NaBH_4_ (50 μL) increasing the absorption to 24%.

B. Mole ratio 2:1 (THTQ:Au(III))

The ama@THT_1_Q[7]Cl (652 μL) was added to H_2_O (1298 μL), and to this, a HAuCl_4_ solution (50 μL) was added. The slightly turbid solution was placed in the microwave reactor and stirred at 70 °C for 15 min, with visible absorption spectrum at λ_max_ = 538 nm. The remaining Au salts were verified by the addition of NaBH_4_ (25 μL) showing an increase of absorption of 11%.

C. Mole ratio 3:1 (THTQ:Au(III))

The ama@THT_1_Q[7]Cl (938 μL) was added to H_2_O (1012 μL), and to this, a HAuCl_4_ solution (50 μL) was added. The solution was placed in the MW reactor and stirred at 70 °C for 15 min at λ_max_ = 539 nm. The absence of the remaining Au salts was verified by the addition of NaBH_4_ (25 μL) showing no increase in absorption.

The conditions of method C were tested for the possible effects of an extended reaction time. Four repeat 15 min cycles gave no significant change in the λ_max_ or absorption.

D. A repeat of method C with ama@THT_1_Q[7]PF_6_

The ama@THT_1_Q[7]PF_6_ was completely soluble in this experiment. A HAuCl_4_ solution (12 μL, 23.9 mM) was added to a solution of ama@THT_1_Q[7]PF_6_ (1990 μL, 0.42 mM ), which immediately formed a pale-yellow turbid mixture. Heating in the MW reactor for 15 min gave a clear colorless solution. A repetition of an immediate 15 min gave no visual change. After resting overnight, a faint red-blue color was observed. Heating at 70 °C for 15 min gave a slight increase in color. Formic acid (90%, 5 μL) was added and the heating repeated 2 times for 15 min, which gave a red solution at λ_max_ = 528 nm.

#### 3.4.2. Preparation AuNP-THT_6_Q[6]

THT_6_Q[6] prepared according to the previous report [33] was determined to have a solubility at RT of <200 mM in pure water and 1 mM in aqueous Ca(OAc)_2_ (50 mM). As the cyclopentylammonium chloride (cpaCl) association complex of THT_6_Q[6] the solubility in pure water is significantly improved. The cpa@ THT_6_Q[6]Cl salt in pure water was soluble to 1.2 mM. A stock solution of HAuCl_4_ (19.3 mM) was used.

A. Reaction in pure water

THT_6_Q[6] (1.5 mg) was suspended in pure water (2 mL) with sonication for 5 min and heating to give a fine cloudy suspension upon cooling. A HAuCl4 (51.5 μL) stock solution was added to form a pale-yellow cloudy solution. The mixture was heated in the MW reactor at 70 °C for 15 min. The pale-yellow suspension gave way to a colorless solution and no AuNP color was evident. Repeating for an additional 15 min gave no significant change. After sitting on the bench for several days, some blue-red color was observed.

B. Reaction in a 50 mM Ca(OAc)_2_ solution

To a solution of THT_6_Q[6] at 0.82 mM in a Ca(OAc)_2_ solution (50 mM, 2 mL) was added to a HAuCl_4_ (51.5 μL) stock solution, which gave a slightly cloudy solution. Heating in the MW reactor at 70 °C for 15 min gave a red solution λ_max_ = 527 nm, and the pH of this solution was 5.5. On other occasions, under identical conditions, 15 min was insufficient with incomplete color and an additional 15 min was applied.

C. Reaction of cpa@THT_6_Q[6]Cl in pure water

The cpa@THT_6_Q[6] was prepared according to the previous report [33] in a solution of water (2 mL). To the completely clear solution, HAuCl_4_ (51.8 μL) was added from the stock solution. The pale-yellow cloudy mixture was heated in the MW reactor at 70 °C for 15 min. The cloudy mixture became clear, with no pale-yellow color remaining. The measured pH was 2. Repeating another 15 min cycle gave no distinct AuNP color. Adding 90% formic acid (100 μL) and repeating the 15 min cycle produced a red solution at λ_max_ = 241 nm.

### 3.5. Purification of AuNP-THTmQ[n]

The AuNP-THTmQ[*n*] solutions were centrifuged at 12,000–20,000 rpm for 10–20 min, and centrifugation time was increased depending on the sample to ensure the sedimentation of a maximum number of particles. The supernatant solution was removed, and the sediment was resuspended in Milli-Q water with 2–10 min sonication. The resuspended samples were verified via visible spectra.

### 3.6. Sulfoxide Affinity for AuNPs

To HAuCl_4_ (211.6 μL, 10 mM stock), diluted with Milli-Q water (1 mL), was added a solution of NaBH_4_ (211.6 μL, 50 mM stock). The mixture of AuNPs was sonicated for 1 min and then purified via centrifugation, as above. The plug of AuNPs was resuspended in a solution of ama@O-THT_1_Q[7]Cl (1 mg) in pure water (1 mL) with the aid of sonication. After 15 min, the solution was centrifuged, the supernatant was removed from the AuNP plug and then resuspended in D_2_O and examined via ^1^H NMR. No sulfoxide resonances were observed.

### 3.7. Control Reaction with Q[7] and ama@Q[7]

The unsubstituted Q[7] and ama@Q[7] were subjected to the same MW reaction conditions as above. Solutions of Q[7] (1990 μL, 1.2 mM) and ama@Q[7]Cl [38] (1960 μL, 1.0 mM), and a stock solution of HAuCl_4_ 23.9 mM were used for the following reactions.

A HAuCl_4_ solution (32.5 μL) was added to a clear solution of Q[7] (1990 μL, 1.2 mM). The solution turned slightly turbid, and the stirred mixture was heated to 70 °C in the MW reactor for 15 min. No color change was observed. After cooling, 90% formic acid (66 μL) was added and the heating in the MW reactor repeated. Again, there was no change.

A HAuCl_4_ solution (27.0 μL) was added to a clear solution of ama@Q[7]Cl [38] (1960 μL, 1.0 mM). The solution turned slightly turbid, and the stirred mixture was heated to 70 °C in the MW reactor for 15 min. The solution was clearer, but no typical AuNP color was observed. After cooling, 90% formic acid (55 μL) was added and the heating in the MW reactor repeated (no change). Extending the reaction time for a further 20 min led to no change. Examination of the reaction mixture via ^1^H NMR also showed no changes in the proton resonances.

## 4. Conclusions

Two examples of tetrahydrothiopheno substituted Q as aqueous solutions were demonstrated to function as reducing agents to Au(I), and in the presence of formic acid, the final step affected the reduction to Au(0) as AuNPs from HAuCl_4_ under MW reactor conditions. In this reaction, a two-step process occurs, whereby the first is where the sulfur of the tetrahydrothiopheno group is oxidized to sulfoxide to form Au(I). The second step is reduction by formic acid to complete the formation of AuNP-THT_m_Q[*n*] conjugates. The association of these conjugates has sufficient affinity so that purification via centrifugation, removal of the supernatant and resuspension enable the isolation of AuNP-THT*m*Q[*n*] detectable via ^1^H NMR spectroscopy. The exact mechanism of the second step reduction is not clear but appears to be dependent on the formation of the Au(I)THTQ complex.

## Data Availability

The data presented in this study are available in article (or Supplementary Materials).

## Supplementary Materials

The following supporting information can be downloaded at: https://www.mdpi.com/article/10.3390/molecules29010168/s1, Figures S1–S10: ^1^H NMR spectra of new compounds, visible spectra of AuNP conjugates of ama@THT_1_Q[7] at varying stages of the reaction, additional TEM micrographs, visible spectra of AuNP conjugate of THT_6_Q[6] and photographs of solutions of AuNP-THT_m_Q[*n*] conjugates.
