# Supplementary material for: Microwave Synthesis of Au Nanoparticles in the Presence of Tetrahydrothiophenocucurbituril
Source: Molecules. 2023 Dec 27;29(1):168. doi: 10.3390/molecules29010168 (PMC10780150; doi:10.3390/molecules29010168)
Supplement: Supplementary file 1 [file molecules-29-00168-s001.zip › molecules-2737059-supplementary.pdf]

Supporting Information

Microwave Synthesis of Au Nanoparticles in the Presence of Tetrahydrothiophenocucurbituril

Asma S. Atthar , Shreya Saha, Ahmed Abdulrahman and Anthony I. Day *

Chemistry, School of Science, University of New South Wales Canberra, Australian Defence Force Academy, Canberra, ACT 2600, Australia;
asmasamaunnisa@gmail.com (A.S.A.); shreya.saha@adfa.edu.au (S.S.);
a.abdulrahman@adfa.edu.au (A.A.)

* Correspondence: a.day@adfa.edu.au.com

Table of Contents:

	Page
Figure S1and S2. The ^{1}H NMR spectrum of ama@THT_1Q[7]Cl in D_2O and The ^{1}H NMR spectrum of ama@O-THT_1Q[7]Cl in D_2O, respectively	**S2**
Figure S3. a) The ^{1}H NMR spectrum of the reaction mixture from the MW reaction of ama@THT_1Q[7] with $HAuCl_4$ in water. b) A zoomed in section of the same sample.	**S3**
Figure S4. ^{1}H NMR spectrum of the reaction mixture from the MW reaction of ama@THT_1Q[7]PF_6 with $HAuCl_4$ after 15 min	**S4**
Figure S5. Visible spectra of AuNP-THTglycoluril prepared in a mixture of DMSO/water (2:1) in the MW reactor over 10 min at 70 °C.	**S5**
Figure S6. Visible spectra of AuNP-ama@THT_1Q[7] prepared in water 1:1 mole ratio of Au(III) to THT_1Q[7], then addition of $NaBH_4$ solution. Figure S7. Visible spectra of AuNP-ama@THT_1Q[7] prepared in pure water in 1:2 mole ratio of Au(III) to THT_1Q[7], then addition of $NaBH_4$ solution.	**S6**
Figure S8. Purified ama@THT_1Q[7] TEM	**S7**
Figure S9. Visible spectrum of AuNP-THT_6Q[6] prepared in an aqueous solution of 50 mM $Ca(OAc)_2$, mole ratio of Au(III) to THT_6Q[6] was 1:1. Figure S10. Photographs of AuNP-THT_mQ[*n*] conjugates	**S8**

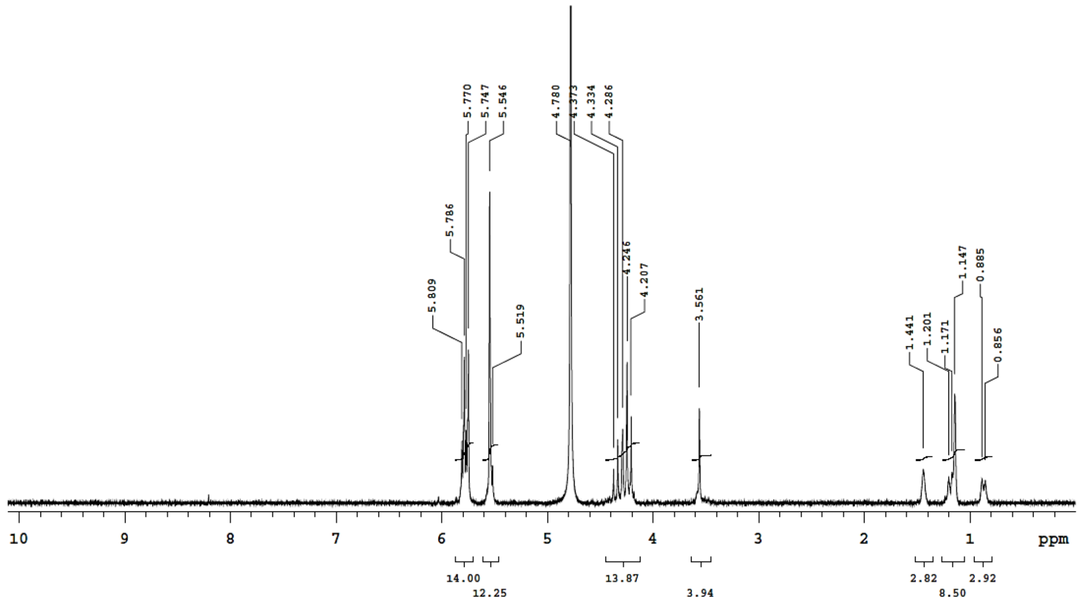

Figure S1. The ^{1}H NMR spectrum of ama@THT$_1$Q[7]Cl in D_2O

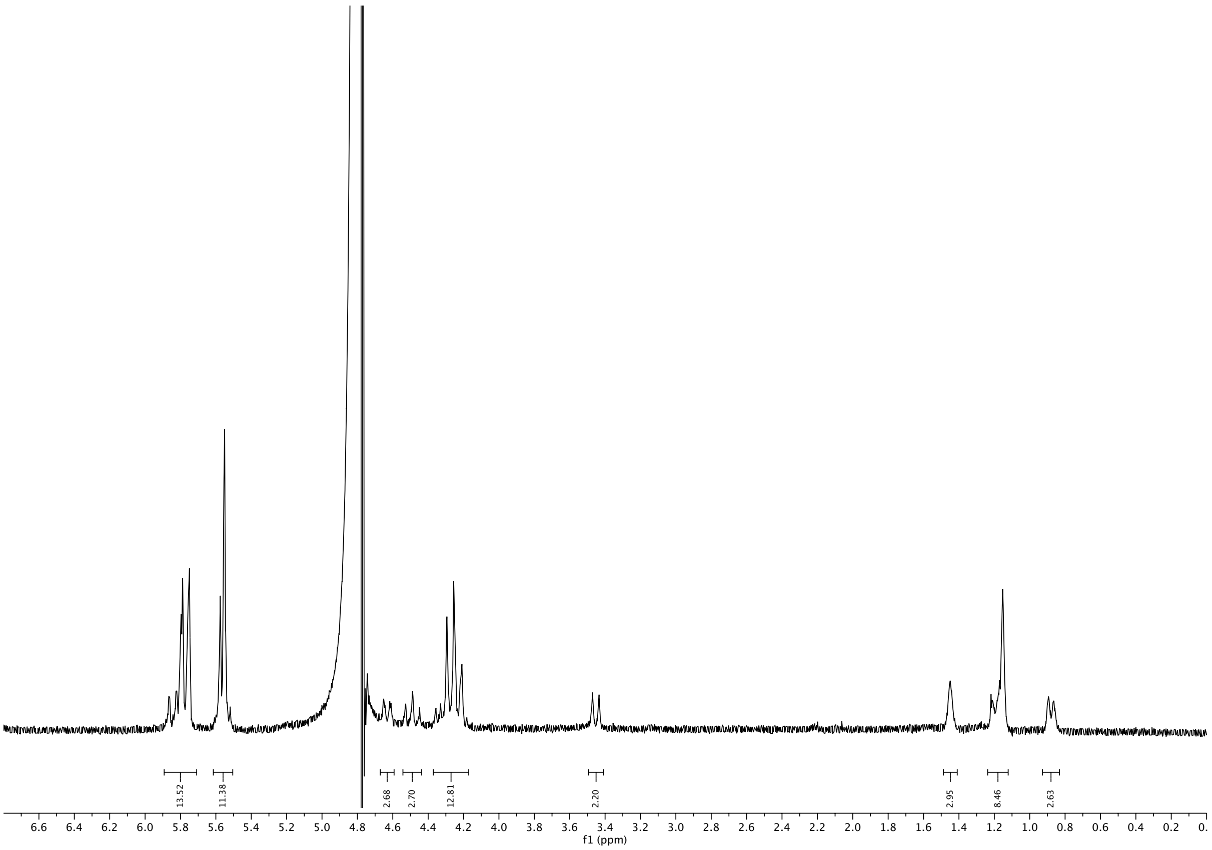

Figure S2. The ^{1}H NMR spectrum of ama@O-THT$_1$Q[7]Cl in D_2O.

a)

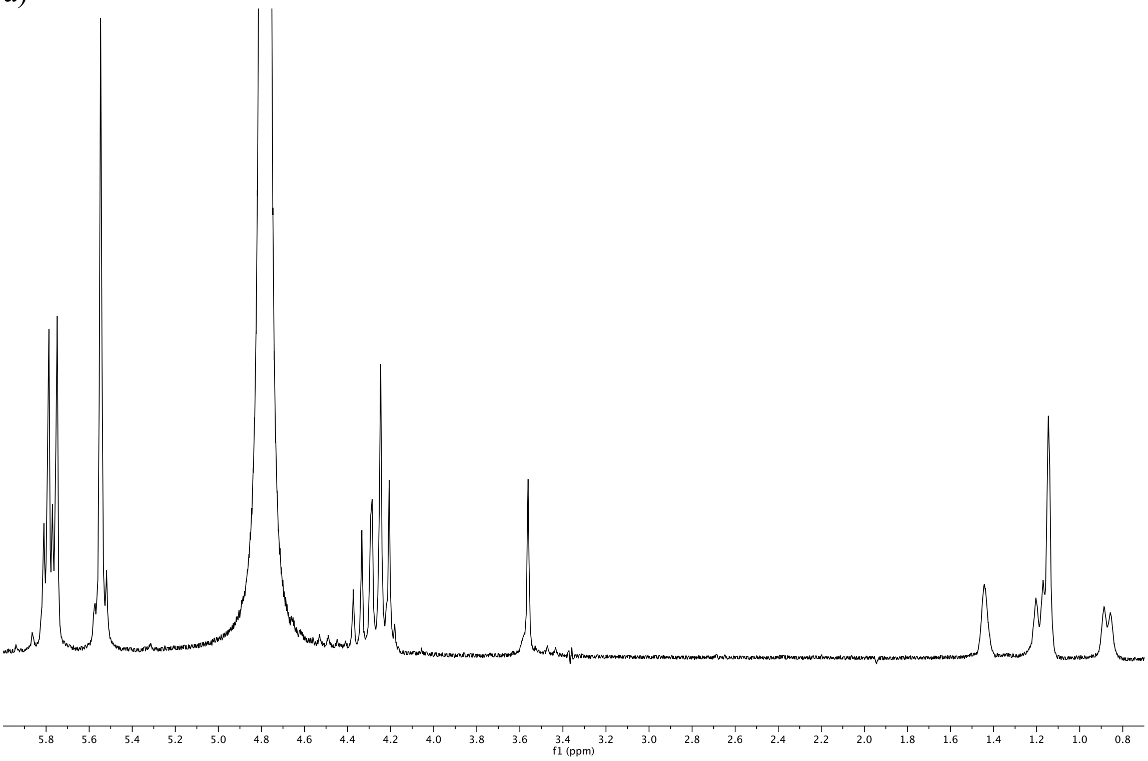

b)

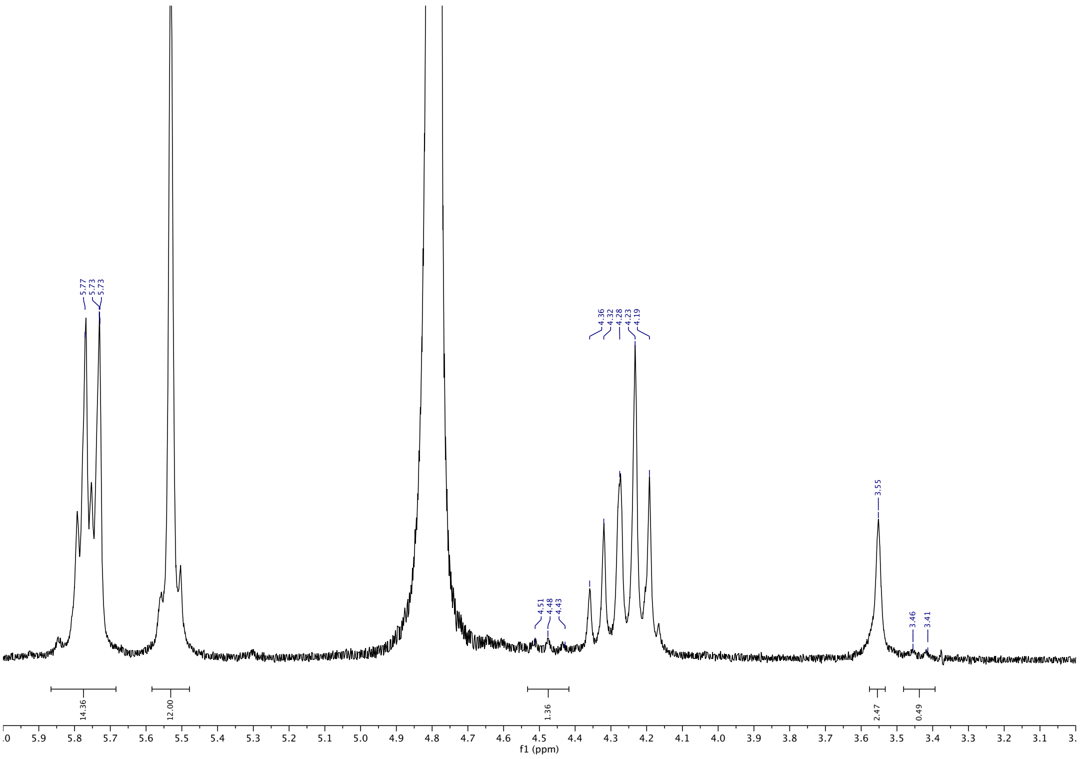

Figure S3. a) The ^{1}H NMR spectrum of the reaction mixture from the MW reaction of ama@$THT_1Q[7]$ with $HAuCl_4$ in water heated to 70 °C for 5 min. A freeze-dried sample dissolved in D_2O. b) A zoomed in section of the same sample.

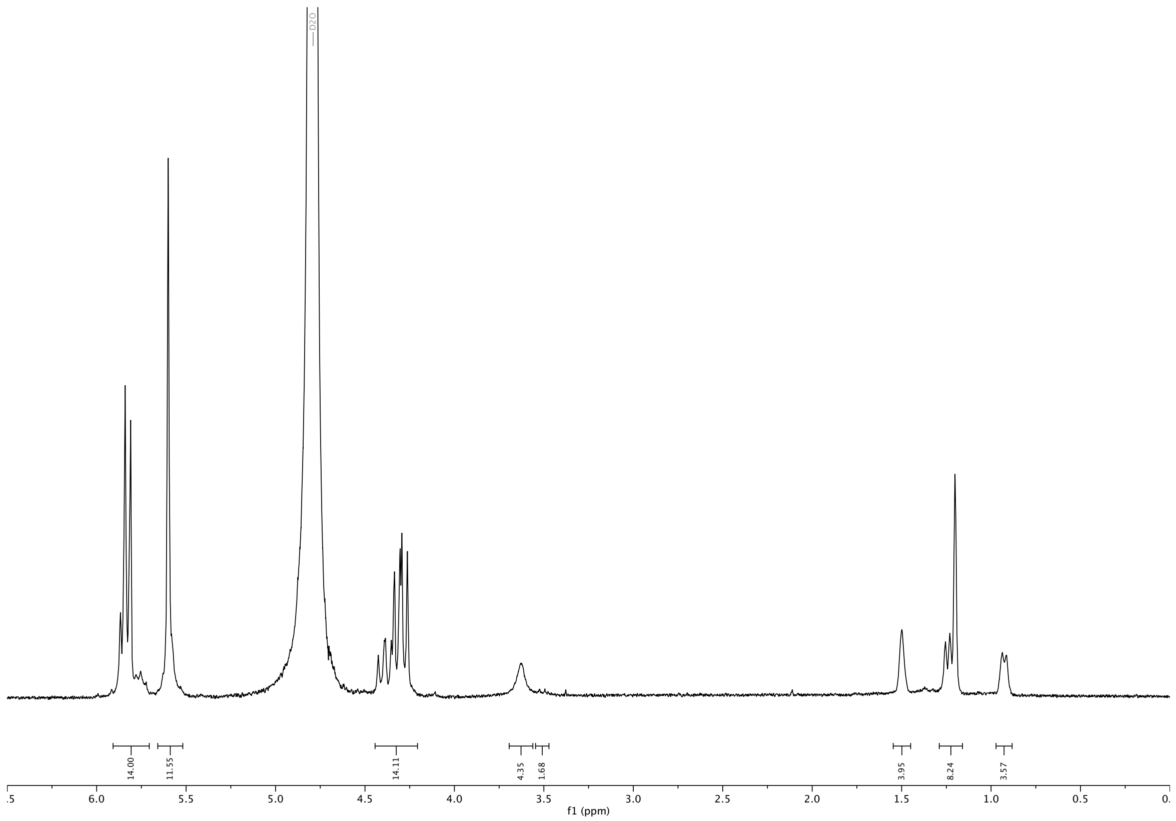

Figure S4. The ^{1}H NMR spectrum (500 MHz) of the reaction mixture from the MW reaction of ama@THT$_1$Q[7]PF$_6$ with HAuCl$_4$ in water heated to 70 °C for 15 min. At this point the solution is colorless and clear.

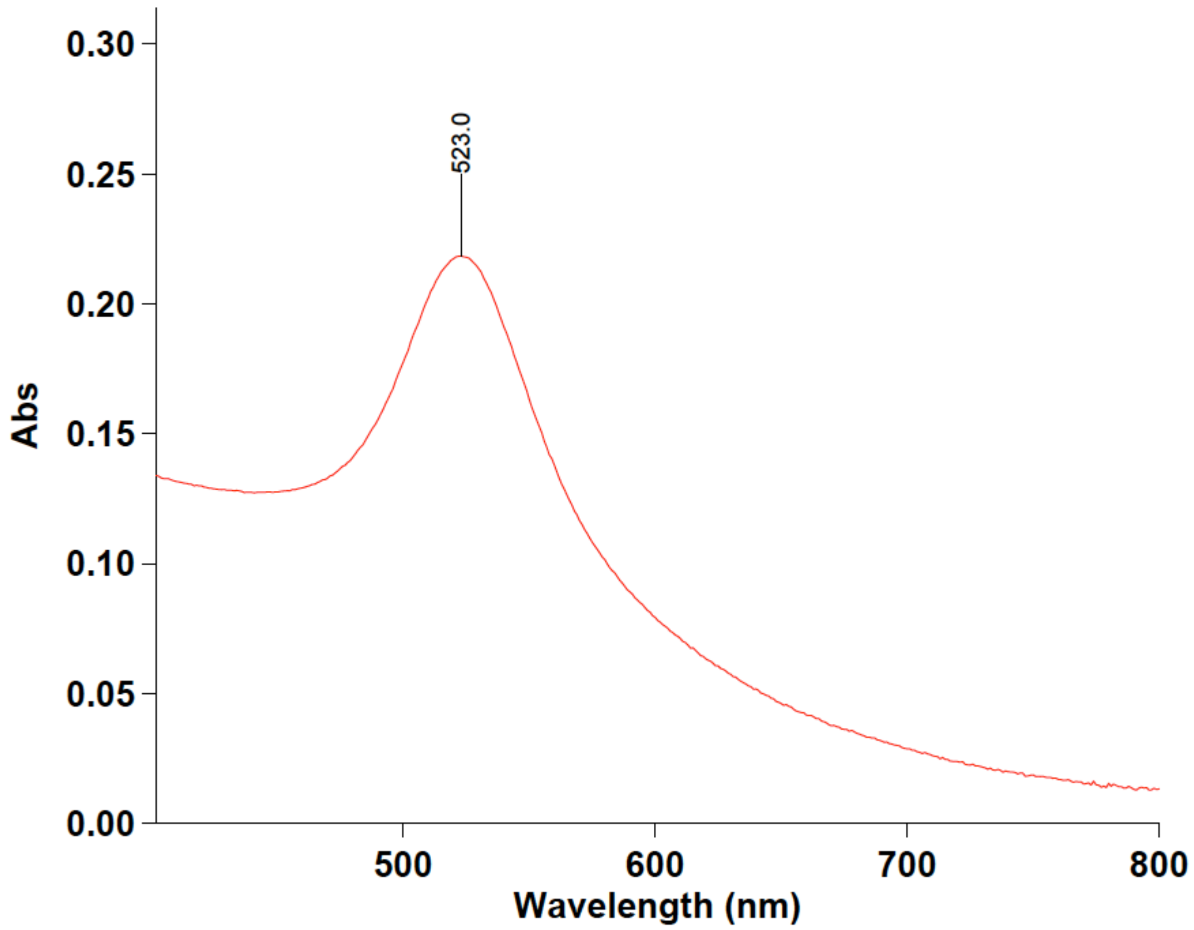

Figure S5. Visible spectra of AuNP-THTglycoluril prepared in a mixture of DMSO/water (2:1) in the MW reactor over 10 min at 70 °C.

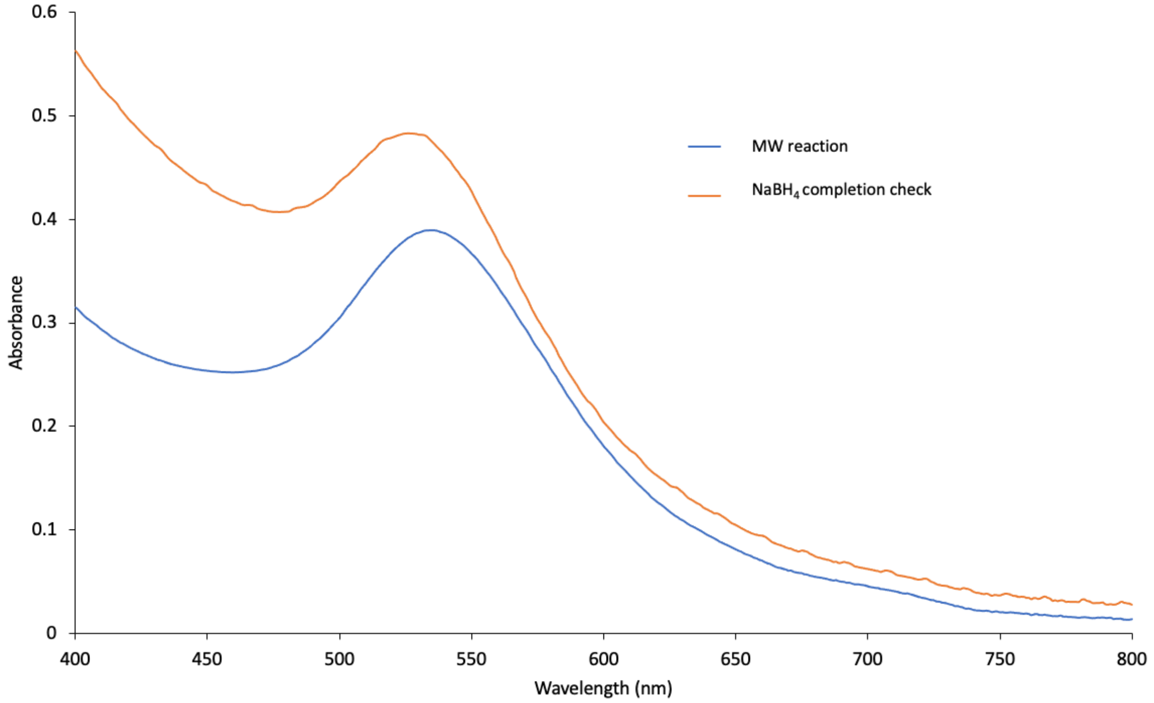

Figure S6. Visible spectra of AuNP-ama@THT$_1$Q[7] prepared in pure water in the MW reactor over 10 min at 70 °C, with a 1:1 mole ratio of Au(III) to THT$_1$Q[7]. After the reaction period was complete, a $NaBH_4$ solution was added revealing the proportion of unreacted Au salts.

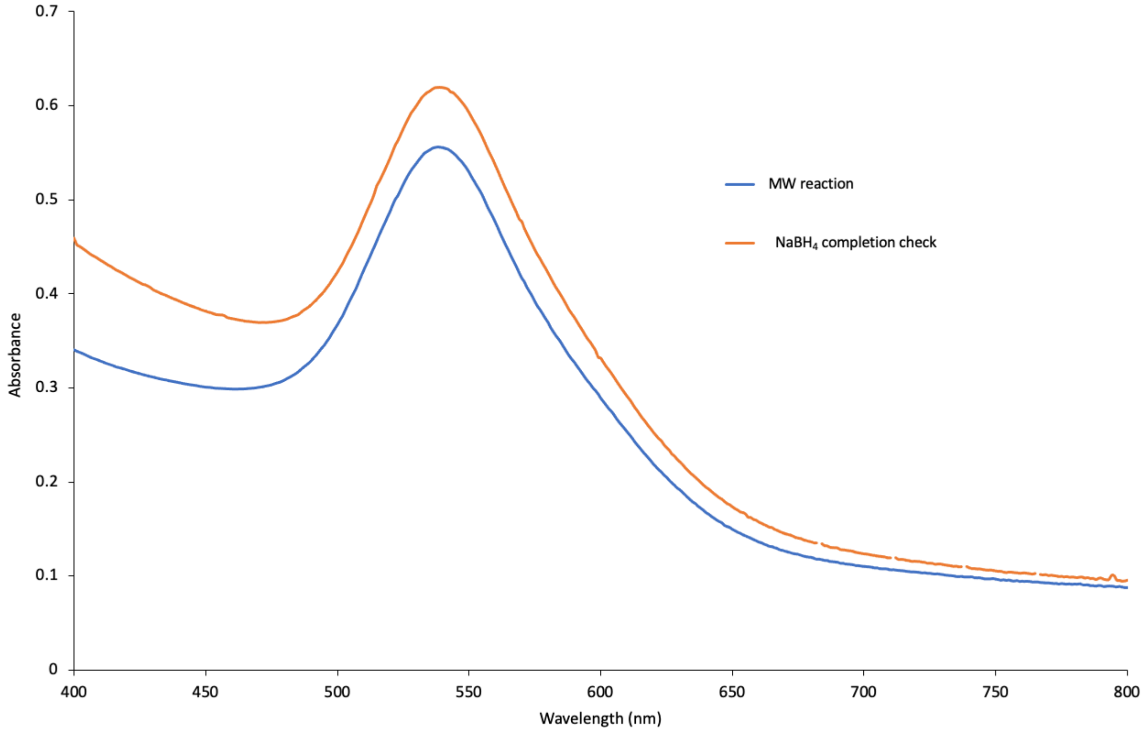

Figure S7. Visible spectra of AuNP-ama@THT$_1$Q[7] prepared in pure water in the MW reactor over 15 min at 70 °C, with a 1:2 mole ratio of Au(III) to THT$_1$Q[7]. After the reaction period was complete, a $NaBH_4$ solution was added revealing the proportion of unreacted Au salts.

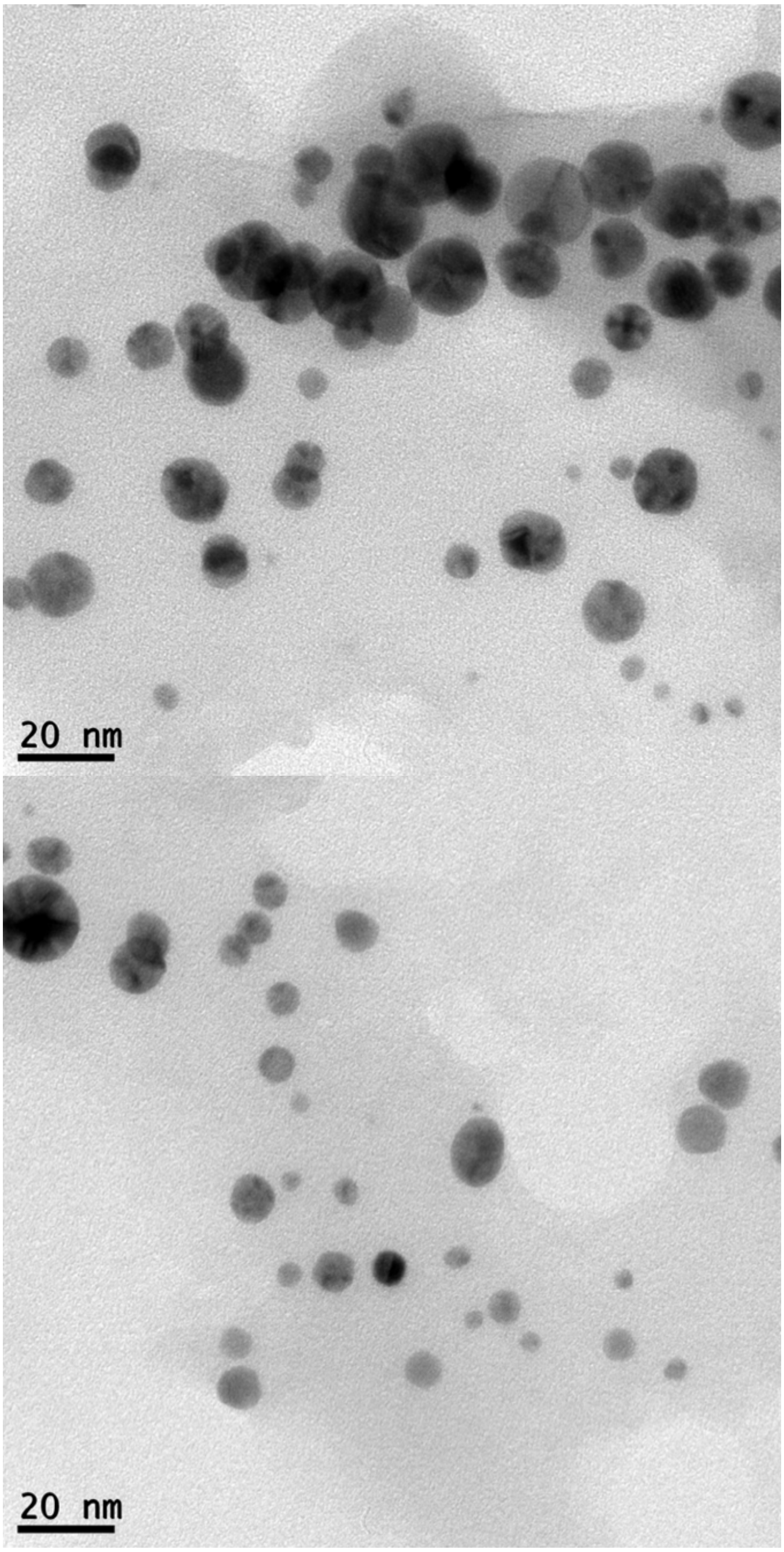

Figure S8. Purified ama@$THT_1Q[7]$ TEM micrographs

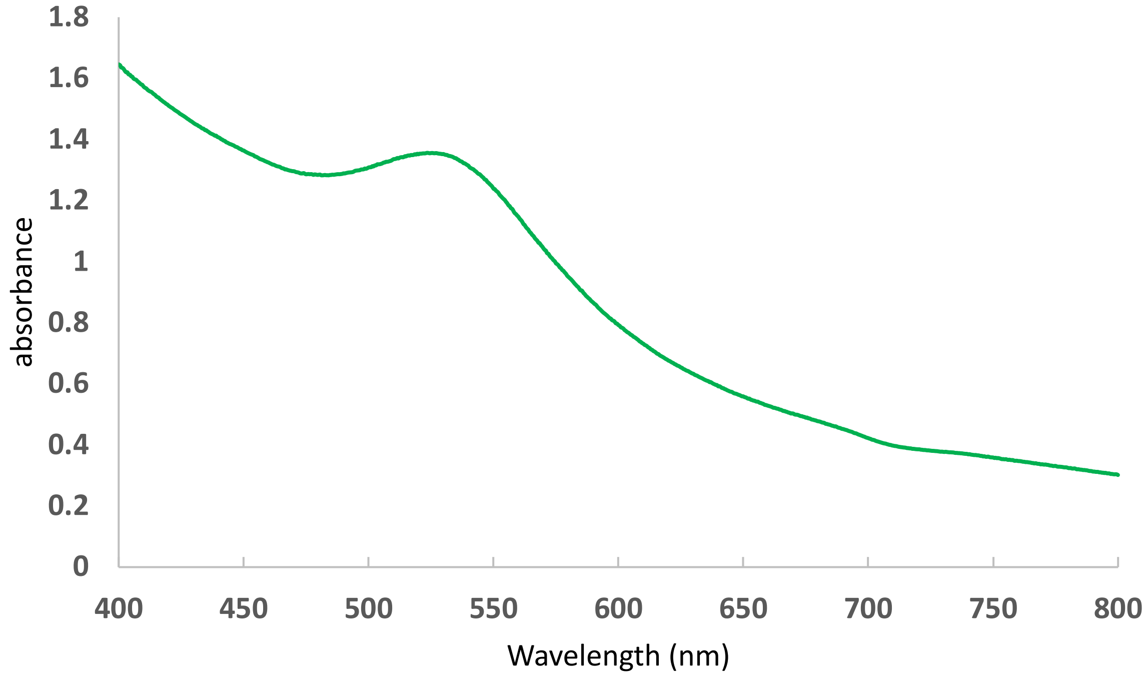

Figure S9. Visible spectrum of AuNP-THT$_6$Q[6] prepared in an aqueous solution of 50 mM $Ca(OAc)_2$ in the MW reactor over 15 min at 70 °C. Mole ratio of Au(III) to THT$_6$Q[6] was 1:1.

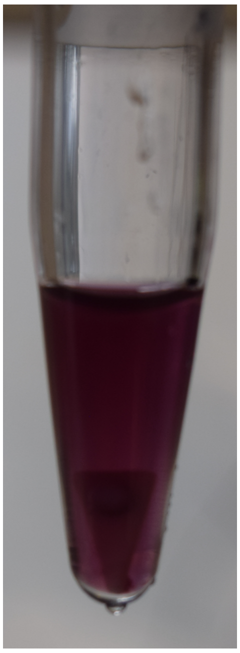

AuNP-THT$_6$Q[6] conjugate in aqueous $Ca(OAc)_2$.

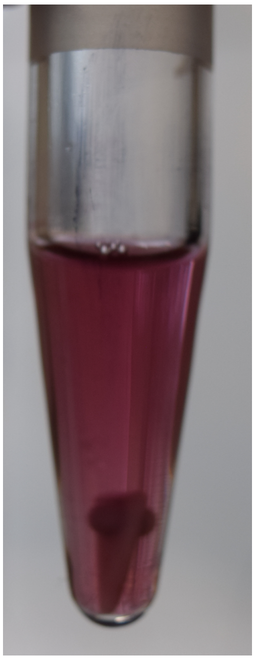

AuNP-ama@THT$_1$Q[7] conjugate in H_2O.

Figure S10. Photographs of AuNP-THT$_m$Q[*n*] conjugates
